# Aging and Sex Influence Cortical Auditory-Motor Integration for Speech Control

**DOI:** 10.3389/fnins.2018.00749

**Published:** 2018-10-17

**Authors:** Jingting Li, Huijing Hu, Na Chen, Jeffery A. Jones, Dan Wu, Peng Liu, Hanjun Liu

**Affiliations:** ^1^Department of Rehabilitation Medicine, The First Affiliated Hospital, Sun Yat-sen University, Guangzhou, China; ^2^Guangdong Work Injury Rehabilitation Center, Guangzhou, China; ^3^Department of Psychology and Laurier Centre for Cognitive Neuroscience, Wilfrid Laurier University, Waterloo, ON, Canada; ^4^Guangdong Province Key Laboratory of Brain Function and Disease, Zhongshan School of Medicine, Sun Yat-sen University, Guangzhou, China

**Keywords:** auditory feedback, speech motor control, aging, sex, event-related potential

## Abstract

It is well known that acoustic change in speech production is subject to age-related declines. How aging alters cortical sensorimotor integration in speech control, however, remains poorly understood. The present event-related potential study examined the behavioral and neural effects of aging and sex on the auditory-motor processing of voice pitch errors. Behaviorally, older adults produced significantly larger vocal compensations for pitch perturbations than young adults across the sexes, while the effects of sex on vocal compensation did not exist for both young and older adults. At the cortical level, there was a significant interaction between aging and sex on the N1-P2 complex. Older males produced significantly smaller P2 amplitudes than young males, while young males produced significantly larger N1 and P2 amplitudes than young females. In addition, females produced faster N1 responses than males regardless of age, while young adults produced faster P2 responses than older adults across the sexes. These findings provide the first neurobehavioral evidence that demonstrates the aging influence on auditory feedback control of speech production, and highlight the importance of sex in understanding the aging of the neuromotor control of speech production.

## Introduction

Speech production is a remarkable motor behavior that involves precisely coordinated movements of multiple muscles and speech articulators, and relies on the integration of sensory feedback into the vocal motor systems (Smotherman, 2007). Auditory feedback is not only essential for the development of speech (Macdonald et al., 2012; Terband et al., 2014); it remains essential for the ongoing maintenance of speech production as evidenced by the rapid compensatory adjustments of vocal motor behavior elicited by unexpected alterations of auditory feedback (Burnett et al., 1998; Houde and Jordan, 1998; Bauer et al., 2006). Neuroimaging studies have shown the event-related potentials (ERPs) of the N1 and P2 responses evoked by vocal pitch errors (Behroozmand et al., 2009; Liu et al., 2011b). These two ERP components are hypothesized to reflect the early detection (i.e., N1) of vocal errors and the later auditory-motor transformation (i.e., P2) necessary to correct for vocal errors during ongoing speech (Behroozmand et al., 2011; Chen et al., 2015). The cortical and subcortical regions involved in this feedback-based control of speech production have also been identified, including auditory- and motor-related areas (e.g., premotor cortex, superior temporal gryus, basal ganglia, and thalamus) as well as fronto-parietal regions (e.g., inferior frontal gyrus, inferior parietal lobule) (Tourville et al., 2008; Zarate and Zatorre, 2008; Tian and Poeppel, 2010; Parkinson et al., 2012; Chang et al., 2013; Behroozmand et al., 2015; Guo et al., 2016). These findings reflect the compensatory mechanisms by which errors in auditory feedback can be detected and corrected to stabilize the production of speech sounds around the desired acoustic targets (Hickok et al., 2011; Tian and Poeppel, 2015).

Most previous investigations of the neural bases of speech motor control have involved young adults, with little attention focused on the effects of aging on auditory-motor integration for speech processing. Considerable evidence has shown that advancing age causes acoustic changes in a number of aspects of speech production (Benjamin, 1981, 1997; Ramig and Ringel, 1983; Mueller, 1997; Sataloff et al., 1997). For example, aging-related changes in voice fundamental frequency (F_0_) through adult life have been well documented (Hollien and Shipp, 1972; Ramig and Ringel, 1983; Decoster and Debruyne, 1997; Mueller, 1997; Sataloff et al., 1997). Older adults exhibit significantly greater instability in their voice F_0_, jitter, and spectral noise and lower vowel formants than young adults (Shipp and Hollien, 1969; Wilcox and Horii, 1980; Ramig and Ringel, 1983; Gorham-Rowan and Laures-Gore, 2006; Torre and Barlow, 2009). Other studies have reported deficits in articulation and prosody with aging, as reflected by decreased speaking rates (Duchin and Mysak, 1987; Wohlert and Smith, 1998) and speech accuracy (Sadagopan and Smith, 2013; Bilodeau-Mercure et al., 2015). The aging-related changes in the peripheral speech mechanisms, in particular in the physiology of the laryngeal system, as well as reduced accuracy of motor control, have been proposed to account for the acoustic changes in speech (Liss et al., 1990; Torre and Barlow, 2009).

Besides, a growing body of literature has revealed the relationship between structural and functional changes in the aging brain and the acoustic changes in aging speech (Eckert et al., 2008; Harris et al., 2009; Soros et al., 2011; Tremblay et al., 2013; Tremblay and Deschamps, 2016; Tremblay et al., 2017). For example, Soros et al. (2011) found greater activation in the inferior frontal gyrus, precentral gyrus, anterior insula, and supplementary motor area in older adults during overt speech production as compared to young adults. During the production of meaningless sequences of speech syllables (e.g., /pa-pa-pa/ vs. /pa-ta-ka/), older adults exhibited significantly longer speech movement time than young adults, and these age-related changes were significantly correlated with structural changes in the bilateral anterior insula, the left primary motor area, the rostral supramarginal gyrus, the right inferior frontal sulcus, and the bilateral striatum (Tremblay and Deschamps, 2016). These findings suggest that the age-related decline in speech production may not be solely the result of a decline in the peripheral speech mechanisms and may instead be related to structural and functional changes in the aging brain (Tremblay et al., 2017). The influence of aging on the neuromotor control of speech production, however, is far from clear.

Given the previously observed changes in speech production with advancing age at the behavioral and neural levels, it is reasonable to assume that the normal aging process may compromise the integration of auditory and motor information, which is necessary for the feedback-based control of speech production. Supportive evidence for this hypothesis comes from two behavioral studies that used a frequency-altered feedback (FAF) paradigm, where older adults produced significantly larger vocal compensations for pitch perturbations they heard than young adults (Liu et al., 2010, 2011c). Despite these behavioral findings, the aging mechanisms of cortical sensorimotor integration in speech processing have received much less attention, leading to the lack of knowledge about the central causes of how normal aging affects auditory-motor integration for speech control. Clarifying the aging effects on speech motor control is not only crucial for our understanding of the relationship between the neurobiology of aging and speech production, but also has important implications for the evaluation and treatment of motor speech disorders caused by a variety of neurological diseases (e.g., Parkinson’s disease, Alzheimer’s disease) that occur most frequently in adults with advancing age.

Note that one important but often overlooked fact is the substantial differences between men and women with regard to the aging process. Men and women differ in terms of the changes in their laryngeal structures with aging, resulting in differential effects of aging on speech production in women vs. men (Linville and Rens, 2001; Gorham-Rowan and Laures-Gore, 2006). For example, voice F_0_ decreases slightly until 50 years of age and then gradually increases afterward for men, while for women voice F_0_ decreases continuously with age or stays constant until menopause after which time it decreases (Decoster and Debruyne, 1997; Sataloff et al., 1997; Torre and Barlow, 2009). As compared to men, women undergo more pronounced laryngeal lowering and vocal tract lengthening across the adult lifespan, leading to differential age-related adjustments of speech production between the sexes (Linville and Rens, 2001). Moreover, considerable evidence has shown the sex-specific differences in the brain structures and functions with aging, as reflected by greater age-related atrophies in the frontal and temporal lobes in men than in women (Cowell et al., 1994; Coffey et al., 1998; Kakimoto et al., 2016). On the other hand, previous studies on young adults have shown the effects of sex on the vocal or cortical ERP responses to pitch feedback errors, where young females produced significantly smaller but faster vocal responses and faster P2 responses to pitch perturbations than young males (Chen et al., 2010; Swink and Stuart, 2012). Therefore, the age-related changes in the laryngeal and brain structures and functions that are involved in speech production are sex-specific, and the differences between men and women should be taken into account in the studies of how the aging process affects speech motor behaviors.

Therefore, the present study investigated the behavioral and neural correlates of age-related auditory-motor control of speech production. Both young and older adults were exposed to unexpected pitch feedback perturbations while producing sustained vowel sounds, and their vocal compensations and cortical ERPs (N1 and P2) were measured and compared across the conditions. In addition to the age, sex was also included as a between-subject factor in the present study. We hypothesized that aging and sex would significantly interact to influence both vocal compensations and cortical ERPs in response to pitch feedback errors, which would reflect a sex-specific aging mechanism that supports auditory feedback control of speech production.

## Materials and Methods

### Subjects

Forty-four native Chinese speakers participated in the present experiment and were assigned to one of two groups according to their age. A young group consisted of 10 male (aged 21–25 years, mean = 22.3 ± 1.6 years) and 12 female (aged 19–25 years, mean = 21.1 ± 1.8 years) adults. An older group consisted of 10 male (aged 60–72 years, mean = 64.7 ± 3.5 years) and 12 female (aged 60–73 years, mean = 64.4 ± 4.1 years) adults. The two groups were matched on sex and language background. Females and males did not differ in their age for both the young (*t* = 1.713, d.f. = 20, *p* = 0.102) and older groups (*t* = 0.171, d.f. = 20, *p* = 0.866). All participants were right-handed and reported no history of speech, hearing, neurological, and mental disorders. All participants but two older participants passed the hearing screening at a threshold of 25 dB HL for pure-tone frequencies of 500, 1000, 2000, and 4000 Hz. The two older participants failed to pass the hearing screening for a pure-tone frequency of 4000 Hz due to their high-frequency hearing loss, but they had no problem perceiving their voice pitch feedback perturbations according to their self-report during the pilot tests. Their data were therefore included in the statistical analyses. Written informed consent was obtained from all participants. The research protocol was in accordance with the Code of Ethics of the World Medical Association (Declaration of Helsinki) and approved by the Institutional Review Board of The First Affiliated Hospital at Sun Yat-sen University of China.

### Apparatus

All participants were tested in a sound-attenuated booth. In order to partially mask the air-born and bone-conducted feedback, we calibrated the recording system by making the intensity of voice feedback heard by participants 10 dB SPL (sound pressure level) higher than that of their voice output. The voice signals were collected using a Genuine Shupu microphone (model SM-306), amplified with a MOTU Ultralite Mk3 Firewire audio interface, and sent to an Eventide Eclipse Harmonizer. A custom-developed software program (Max/MSP, v.5.0 by Cycling 74) was used to control the Harmonizer to pitch-shift the amplified voice signals. This program also generated transistor–transistor logical (TTL) pulses to mark the onset of each pitch shift. Finally, the pitch-shifted voice signals were amplified by an ICON NeoAmp headphone amplifier and fed back to participants through insert earphones (ER1-14A, Etymotic Research Inc.). The vocal output and feedback signals as well as the TTL pulses were collected by a PowerLab A/D converter (model ML880, AD Instruments, Castle Hill, NSW, Australia) at a sampling frequency of 10 kHz, and recorded using LabChart software (v.7.0 by AD Instruments).

The electroencephalograph (EEG) signals were collected with the voice signals simultaneously using a 64-electrode Geodesic Sensor Net (Electrical Geodesics Inc., Eugene, OR, United States). The EEG signals that were referenced to the vertex (Cz) (Ferree et al., 2001) were amplified by a Net Amps 300 amplifier (Z_in_≈200 MΩ; Electrical Geodesics Inc.) and recorded with a sampling frequency of 1000 Hz using NetStation software (v.4.5, Electrical Geodesics Inc.). The TTL pulses were sent to the EEG recording system via a DIN cable. Given that the Net Amps 300 amplifier accepts scalp-electrode impedances up to 40–60 kΩ, the impedance levels of individual sensors were adjusted and maintained below 50 kΩ throughout the recording (Ferree et al., 2001).

### Procedure

All participants were instructed to sustain a vowel sound /u/ for about 4–5 s at their conversational voice pitch and loudness. During each vocalization, participants heard their voice pseudo-randomly pitch-shifted + 200 or + 500 cents (100 cents = 1 semitone) 4–5 times. In order to reduce participants’ expectancy of the pitch perturbations, we presented the first pitch perturbation with a delay of 500–1000 ms after the vocal onset and the succeeding pitch perturbations with an inter-stimulus interval (ISI) of 700–900 ms. The duration of each pitch perturbation lasted 200 ms. Liu et al. (2011c) reported that, regardless of the direction of pitch perturbation, larger vocal compensations for older adults aged 61–75 years than for young adults aged 19–30 years were found in the 100 cents condition but absent in the 50 cents condition. Also, our preliminary tests showed that older adults had difficult perceiving small pitch perturbations (100 cents or smaller). Thus, all participants were exposed to pitch perturbations of +200 and +500 cents in the present study. Participants produced 40–50 consecutive vocalizations, resulting in a total of about 100 trials that were +200 cents in size and 100 trials that were +500 cents trials.

### Vocal Data Analysis

The voice signals were analyzed off-line using a custom-developed software program (IGOR PRO, v.6.0, Wavemetrics, Inc., Lake Oswego, OR, United States). First, voice F_0_ contours in Hz were extracted using Praat software (Boersma, 2001) and then were converted to the cents scale using the following formula: cents = 100 × (12 × log2(F_0_/reference)) [reference = 195.997Hz (G3 note)]. Next, the voice F_0_ contours in cents were segmented into epochs ranging from 200 ms before to 700 ms after the onset of the pitch shift and submitted to a visual inspection procedure to reject trials with vocal interruptions or signal processing errors. Overall, 78% of trials were regarded as artifact-free trials and submitted to the averaging procedure. Finally, these artifact-free trials were averaged and baseline-corrected to generate an overall response for each condition. The magnitude and latency of an overall vocal response were defined as the amplitude and time of the greatest F_0_ value following the response onset.

### EEG Data Analysis

NetStation software was used for the off-line analyses of the EEG signals. First, a band-pass filter with cut-off frequencies of 1–20 Hz was used to filter the EEG signals. The filtered EEG signals were then segmented into epochs with a window of 200 ms before and 500 ms after the onset of the pitch perturbation. The segmented epochs were submitted to an artifact detection procedure, during which trials that included voltage values that exceeded ±55 μv of the moving average over an 80-ms window were excluded from further analysis. Individual electrodes that contained artifacts in more than 20% of the epochs and files that contained more than 10 bad channels were excluded from the averaging procedure. Overall, 82% of trials were retained for the following analysis. After re-referencing to the average of the electrodes on each mastoid, artifact-free trials were averaged and baseline-corrected to generate an overall ERP response for each condition. The amplitudes and latencies of the N1 and P2 components were extracted as the negative and positive peaks in the time windows of 80–180 ms and 160–280 ms after the onset of the pitch perturbation from 10 electrodes (FC1, FC2, FC3, FC4, FCz, C1, C2, C3, C4, and Cz). They were chosen because cortical ERPs to pitch perturbations are mostly prominent at frontal and central electrodes (Chen et al., 2012; Scheerer et al., 2013a).

### Statistical Analysis

The values of vocal and ERP responses to pitch-shifted auditory feedback were subject to repeated-measures analyses of variance (RM-ANOVAs) in SPSS (v.20.0). The magnitudes and latencies of compensatory vocal responses were analyzed using three-way RM-ANOVAs, in which stimulus (+200 and +500 cents) was chosen as a within-subject factor while age (young and older adults) and sex (female and male) were chosen as between-subject factors. The amplitudes and latencies of N1 and P2 responses were analyzed using four-way RM-ANOVAs, including two within-subject factors of stimulus magnitude and electrode site (FC1, FC2, FC3, FC4, FCz, C1, C2, C3, C4, and Cz) and two between-subject factors of age and sex. Significant higher-order interactions between any of those variables led to subsidiary RM-ANOVAs. *Post hoc* analyses were performed using Bonferroni adjustment for multiple comparisons. A violation of the sphericity assumption resulted in a correction of probability values for multiple degrees of freedom. An alpha level of 0.05 was accepted as a level of significance.

## Results

### Behavior Findings

**FIGURE 1:**
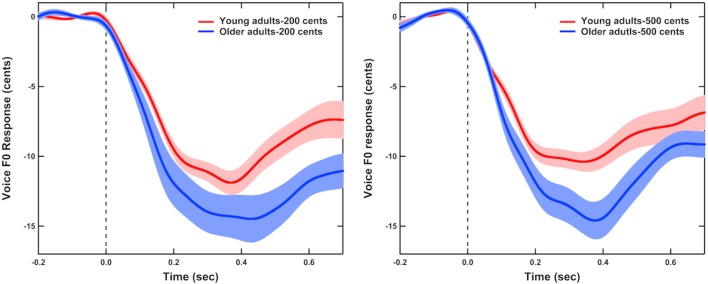
Grand-averaged voice F_0_ contours in response to pitch perturbations of +200 **(left)** and +500 cents **(right)** produced by young (red) and older adults (blue). Highlighted areas represent the standard errors of the mean vocal responses, and the vertical dashed lines represent the onset of the pitch perturbation.

**FIGURE 2:**
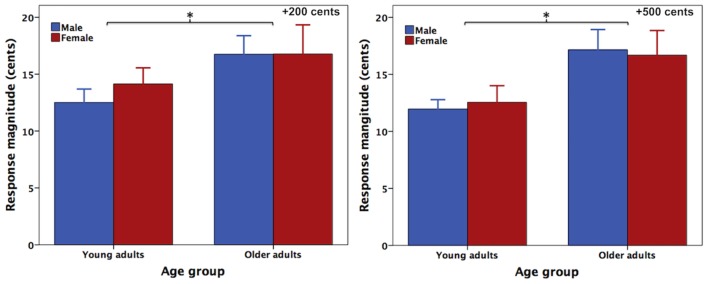
T-bar plots (means and standard errors) of the absolute values of compensatory vocal responses to pitch perturbations of +200 **(left)** and +500 cents **(right)** produced by female (red) and male (blue) speakers as a function of age. The asterisks indicate that older adults produced significantly larger vocal compensations for pitch perturbations than young adults across the sexes and stimuli.

**Figure 1** shows the grand-averaged voice F_0_ contours in response to +200 (left) and +500 cents (right) pitch perturbations produced by young and older adults, indicating the aging effects on the compensatory vocal responses. One three-way RM-ANOVA conducted on the magnitudes of vocal compensations revealed a significant main effect of age [*F*(1,40) = 5.666, *p* = 0.022], showing that older adults (16.8 ± 6.8 cents) produced significantly larger vocal compensations than young adults (12.8 ± 4.2 cents) (see **Figure 2**). The main effects of stimulus [*F*(1,40) = 0.847, *p* = 0.363] and sex [*F*(1,40) = 0.067, *p* = 0.797] (see **Figure 2**) as well as the interactions among these variables (*p* > 0.2), however, did not reach significance. In addition, the latencies of vocal compensations measured as the peak time of vocal response magnitude did not vary as a function of age [*F*(1,40) = 0.028, *p* = 0.867], sex [*F*(1,40) = 0.508, *p* = 0.480], and stimulus [*F*(1,40) = 0.169, *p* = 0.683]. There were also no significant interactions among these variables (*p* > 0.1).

### ERP Findings

**FIGURE 3:**
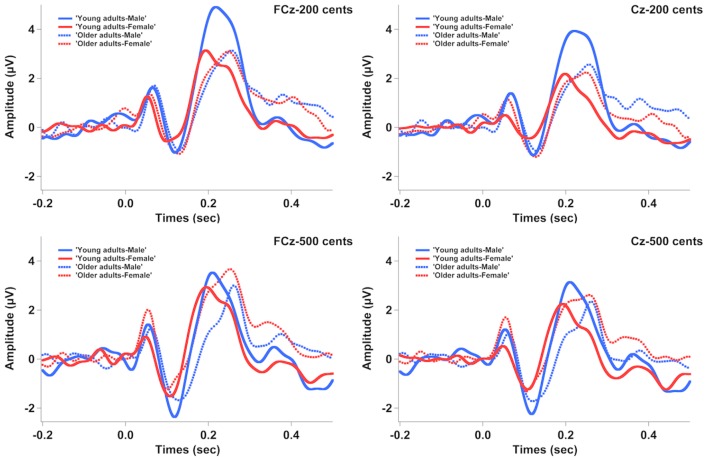
Grand-averaged ERP waveforms in response to pitch perturbations of +200 **(top)** and +500 cents **(bottom)** as a function of age and sex. The solid red and blue lines represent the ERP responses produced by young females and young males, while the dashed red and blue lines represent the ERP responses produced by older females and older males.

**FIGURE 4:**
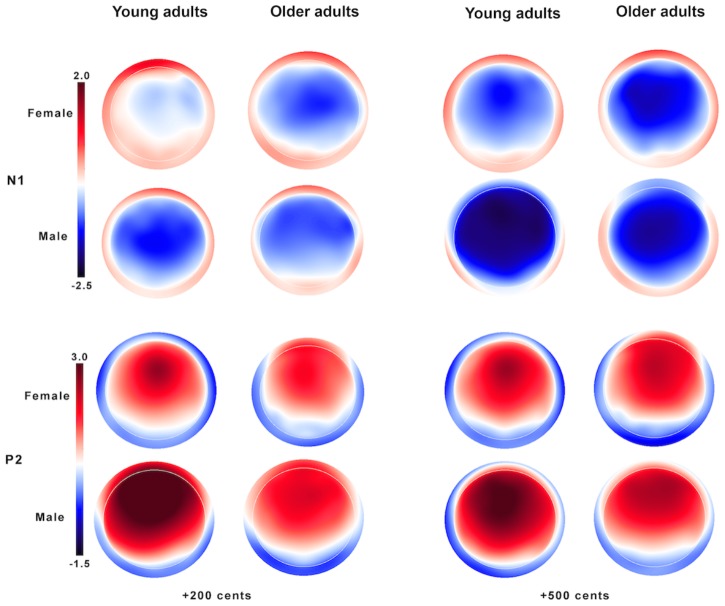
Topographical distributions of the N1 **(top)** and P2 amplitudes **(bottom)** in response to pitch perturbations of +200 **(left)** and +500 cents **(right)** as a function of age and sex.

**FIGURE 5:**
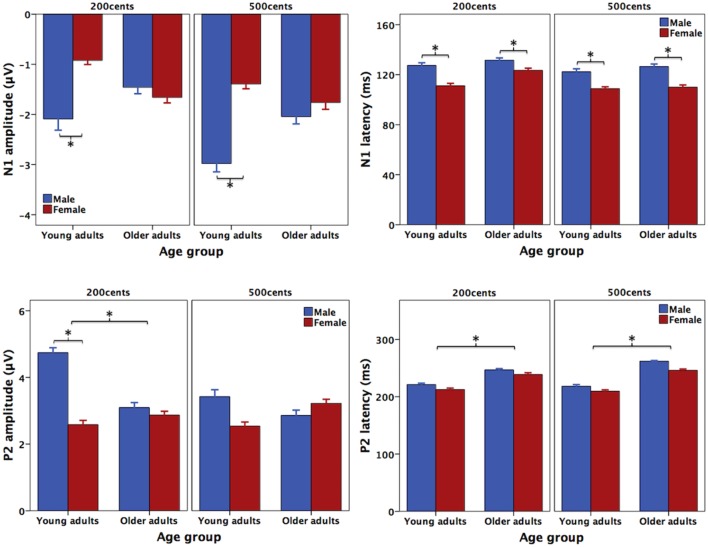
T-bar plots (means and standard errors) of the amplitudes and latencies of N1 **(top)** and P2 **(bottom)** responses to pitch perturbations of +200 and +500 cents produced by female (red) and male (blue) speakers as a function of age. The asterisks indicate significant differences across the conditions.

**Figure 3** shows the grand-averaged ERP waveforms in response to +200 (top) and +500 cents (bottom) pitch perturbations as a function of age and sex, showing that age and sex interact to influence the N1 and P2 responses. Similar trends were also observed in **Figure 4** showing the topographical distributions of the N1 and P2 amplitudes across the conditions. One four-way RM-ANOVA conducted on the N1 amplitudes revealed that the +500 cents condition was associated with significantly larger (more negative) N1 magnitudes than the +200 cents condition [*F*(1,40) = 8.421, *p* = 0.006]. The main effects of age [*F*(1,40) = 0.086, *p* = 0.771] and site [*F*(9,360) = 2.140, *p* = 0.096] did not reach significance, but the main effect of sex [*F*(1,40) = 3.282, *p* = 0.078] and the interaction between age and sex [*F*(1,40) = 2.934, *p* = 0.094] were marginally significant. Considering theoretical motivations can justify conducting *post hoc* analyses for each condition (Maxwell and Delaney, 2004), we performed two three-way RM-ANOVAs on young and older adults to examine the effects of sex on the N1 amplitudes. The results revealed significantly larger N1 amplitudes for males than for females in young adults [*F*(1,40) = 5.515, *p* = 0.029] (see **Figure 5**), whereas such sex effects did not exist in older adults [*F*(1,40) = 0.006, *p* = 0.941].

As for the N1 latencies, the +500 cents condition elicited significantly faster N1 responses than the +200 cents condition [*F*(1,40) = 7.254, *p* = 0.010], and females produced significantly faster N1 responses than males [*F*(1,40) = 7.072, *p* = 0.011] (see **Figure 5**). There was also a significant main effect of site [*F*(1,40) = 3.821, *p* = 0.013] as a result of significantly shorter N1 latencies associated with electrode FCz relative to FC4 and C4. The main effect of age [*F*(1,40) = 1.159, *p* = 0.288] as well as the interactions among these factors (*p* > 0.2) did not reach significance.

One four-way RM-ANOVA conducted on the P2 amplitudes revealed a significant main effect of site [*F*(9,360) = 39.531, *p* < 0.001] and marginally significant main effects of stimulus [*F*(1,40) = 3.921, *p* = 0.055] and sex [*F*(1,40) = 7.254, *p* = 0.075]. Although the main effect of age did not reach significance [*F*(1,40) = 0.610, *p* = 0.439], significant interactions were found between stimulus and age [*F*(1,40) = 5.523, *p* = 0.024] and between stimulus and sex [*F*(1,40) = 8.852, *p* = 0.005]. A subsequent three-way RM-ANOVA for the +200 cents condition showed a significant main effect of sex [*F*(1,40) = 9.219, *p* = 0.004], as well as a significant interaction between age and sex [*F*(1,40) = 6.042, *p* = 0.018], but the main effect of age did not reach significance [*F*(1,40) = 2.985, *p* = 0.092]. Follow-up two-way RM-ANOVAs revealed significantly larger P2 amplitudes for males than for females in young adults [*F*(1,20) = 14.643, *p* = 0.001], but no sex effect existed in older adults [*F*(1,20) = 0.173, *p* = 0.682] (see **Figure 5**). As well, older adults produced significantly smaller P2 amplitudes than young adults for males [*F*(1,18) = 6.668, *p* = 0.019] but not for females [*F*(1,18) = 0.352, *p* = 0.559] (see **Figure 5**). By contrast, another three-way RM-ANOVA for the +500 cents condition showed that the main effects of age [*F*(1,40) = 0.017, *p* = 0.898] and sex [*F*(1,40) = 0.319, *p* = 0.575] as well as their interactions [*F*(1,40) = 1.852, *p* = 0.181] did not reach significance.

As for the P2 latencies, young adults produced faster P2 responses than older adults [*F*(1,40) = 29.391, *p* < 0.001] (see **Figures 3**, **5**). The main effects of sex [*F*(1,40) = 2.897, *p* = 0.097], stimulus [*F*(1,40) = 1.382, *p* = 0.247], and site [*F*(1,40) = 1.951, *p* = 0.104] as well as the interactions among these factors (*p* > 0.2), however, did not reach significance.

## Discussion

The present study examined the behavioral and cortical correlates of auditory-motor integration for vocal pitch regulation as a function of age and sex. The behavioral results showed that older adults exhibited significantly larger vocal compensations for pitch perturbations than young adults across the sexes. Age and sex, however, interacted significantly to influence the cortical N1 and P2 responses. Generally, older males produced significantly smaller P2 amplitudes than young males, while young males produced significantly larger N1 and P2 amplitudes than young females. In addition, females exhibited shorter N1 latencies than males regardless of age, while young adults exhibited shorter P2 latencies than older adults across the sexes. These findings provide neurobehavioral evidence that demonstrates the effects of aging and sex on auditory feedback control of vocal pitch, suggesting that normal aging influences the cortical mechanisms that support auditory-motor integration for speech control in a sex-specific manner.

Our behavioral findings are consistent with the previously reported results that showed significantly larger vocal compensations for pitch perturbations produced by older adults relative to young adults (Liu et al., 2010, 2011c). By contrast, professional singers who are skilled at precise vocal control produced significantly smaller vocal compensations than non-musicians (Jones and Keough, 2008) and even completely ignored vocal pitch perturbations (Zarate and Zatorre, 2008). In light of the hypothesis that a partial correction of sensory feedback errors allows the audio-vocal system to stabilize the online control of speech production around the desired level (Houde and Nagarajan, 2011), enhanced vocal compensations in older adults are suggestive of reduced auditory-motor control of speech production with normal aging. In addition, we did not find the sex effects on vocal compensations for both young and older adults, which is in line with a behavioral study on young adults by Scheerer et al. (2013b). By contrast, Chen et al. (2010) reported that young males produced larger vocal compensations than young females. This inconsistency could be related to the differences in methodology such as the magnitude/direction of pitch perturbations.

More importantly, we found significant interactions between aging and sex on the cortical processing of auditory feedback errors during vocal pitch regulation. With advancing age, P2 latencies became significantly slower across the sexes, while P2 amplitudes became significantly smaller for males. To the best of our knowledge, this is the first electrophysiological evidence that demonstrates the effects of aging on auditory feedback control of speech production, providing further support to the contributions of structural and functional changes in the aging brain to age differences in speech production (Soros et al., 2011; Tremblay et al., 2013, 2017; Tremblay and Deschamps, 2016). With respect to the sex effects, young males produced significantly larger N1 and P2 amplitudes than young females, which is in line with one sex-specific developmental study of speech motor control by Liu et al. (2013). In addition, females exhibited shorter N1 latencies than males regardless of age. Similar results were also reported in previous studies on young adults (Swink and Stuart, 2012; Scheerer et al., 2013b). These findings suggest that aging and sex may interact significantly to influence the cortical auditory-motor mechanisms of speech production. Note that the aging effects on the P2 responses were found in the +200 cents condition but were absent in the +500 cents condition. Previous research has suggested differential mechanisms underlie the auditory-motor processing of small pitch perturbations (e.g., less than 200 cents) that are perceived as self-produced speech errors and large pitch perturbations (e.g., 400 cents or more) that are perceived as externally generated sounds (Burnett et al., 1998; Hain et al., 2000; Behroozmand and Larson, 2011). Thus, our results may reflect aging-related differences in the cortical processing of pitch feedback errors of one’s own voice.

Aging-related acoustic changes in speech production have been attributed to physiological changes in laryngeal structures with age (e.g., vocal fold atrophy, degradation of tissue, glottal incompetence) (Ramig et al., 2001; Gorham-Rowan and Laures-Gore, 2006; Torre and Barlow, 2009). Previous research has demonstrated groups of laryngeal muscles in regulating voice F_0_ through the electromyography (EMG) recording (Hirano et al., 1970; Ludlow et al., 1992; Liu et al., 2011a). For example, the falsetto register produced ctricothyroid and thyroarytendoid EMG responses that either decreased or increased along with the corresponding vocal compensations for pitch perturbations (Liu et al., 2011a). It is reasonable to assume that a decline in the precise control of voice F_0_ that results from aging-related changes to laryngeal structures may result in enhanced vocal responses to pitch feedback errors. An alternative hypothesis that may also explain our behavioral results is that the interaction between kinesthetic and auditory feedback changes with normal aging. Larson et al. (2008) found significantly larger vocal compensations for pitch perturbations when the vocal fold mucosa was anesthetized as compared to the pre-anesthetic condition, and proposed that auditory feedback interacts with kinesthetic feedback to determine voice F_0_. Auditory feedback becomes predominant in speech motor control as a result of decreased kinesthetic feedback, and vice versa. Given that the function of kinesthetic feedback is interfered or impaired due to aging-related changes to laryngeal structures (Gorham-Rowan and Laures-Gore, 2006), it is plausible that older adults may weight auditory feedback more heavily to detect mismatches between intended and actual vocal output, thereby producing larger vocal compensations as compared to young adults.

The findings that older adults exhibited slower and smaller cortical P2 responses to pitch perturbations relative to young adults, however, suggest that age differences in speech motor control may also involve a change in cortical auditory-motor processing of speech. Considering that advanced age is characterized by deficits of cognitive functions such as working memory and executive control (Salthouse, 1996; Park et al., 2002) and inhibitory control is a frontally mediated cognitive function to suppress reflex-like or inappropriate behavioral responses (Burle et al., 2004) that deteriorates during aging (Nielson et al., 2002), we hypothesize that enhanced vocal compensations observed in older adults may be the result of a deficit in the top-down mechanism that supports speech motor control (Tian and Poeppel, 2012). In a recent study by Guo et al. (2017), participants exhibited suppressed vocal compensations for pitch perturbations that were correlated with improved working memory capacity and enhanced P2 responses that were predicted by pre-training working memory capacity in the fronto-parietal regions. Since working memory is closely interrelated with inhibitory control (Chmielewski et al., 2015), Guo et al. (2017) proposed that working memory may generate an inhibitory influence on vocal adjustment to prevent vocal production from being excessively influenced by auditory feedback. Impairment of this top–down inhibitory mechanism may lead to excessive vocal compensations for feedback errors, as evidenced by the findings that patients with Alzheimer’s disease (AD) produced enhanced vocal compensations that were correlated with executive dysfunction and reduced compensation durations that were correlated with memory dysfunction (Ranasinghe et al., 2017). Therefore, the top–down inhibitory mechanism that involves in speech motor control may decline with advancing age as reflected by decreased amplitudes and prolonged latencies of P2 responses, leading to a failure of inhibiting the influence of feedback errors that causes increased vocal compensations.

It is noteworthy that aging-related cortical responses to pitch perturbations varied as a function of sex. Specifically, aging-related decreases in the P2 amplitudes occurred in males but not in females, and males produced significantly larger N1 and P2 responses than females in young adults but not in older adults. Thus, the sex difference in the cortical responses to vocal pitch errors was manifested in adulthood but became insignificant as age advanced, reflecting aging-related changes in cortical auditory-motor control of speech production in a sex-specific manner. These findings may be related to the differences in the progressive changes in brain structures and functions between men and women. For example, age-related increase in the lateral fissure cerebrospinoal fluid volume, a marker of frontotemporal atrophy, was significantly greater in men than in women (Coffey et al., 1998). And, as compared to women, men were associated with more atrophic changes to the frontal cortex (Kakimoto et al., 2016). Alternatively, sex hormone changes across the menstrual cycle or after menopause may also contribute to the sex effects on speech motor control. Zhu et al. (2016) found that young females produced larger vocal compensations when estradiol levels were low during the menstrual phase and smaller P2 amplitudes when progesterone levels were high during the luteal phase. In addition, females with high estradiol levels produced significantly smaller mismatch negativity amplitudes in response to unattended changes in speech prosody than men with low estrogen (Schirmer et al., 2008). Therefore, sex hormone data may have to be evaluated in order to elucidate the sex-specific aging of sensorimotor integration for speech control.

There are two primary limitations in the present study that should be acknowledged. One is the small sample size obtained for analyses in the present study, resulting in low statistical power. Larger sample sizes will be needed to test whether the observed influences of aging and sex on speech motor control are reproducible. On the other hand, the proposed explanation that the top–down inhibitory mechanisms contribute to aging-related changes in speech motor control remains speculative, given the lack of cognitive measures for all participants. Future research should include a comprehensive assessment of cognitive functions such as working memory, executive function, and attention in order to provide evidence of validity.

## Conclusion

The present study provides neurobehavioral evidence that demonstrate the sex-specific aging process of auditory-motor integration for speech control. As compared to young adults, older adults produced significantly larger vocal compensations for pitch feedback errors and slower cortical P2 responses. An interaction between aging and sex was found, as reflected by smaller P2 amplitudes for older males than for young males and larger N1 and P2 amplitudes for young males than for young females. These findings suggest that cortical mechanisms that support auditory feedback control of speech production can be influenced by normal aging, and that sex should be considered essential in understanding the aging of speech motor control.

## Author Contributions

HL and PL designed the experiments. JL, HH, NC, and DW performed the experiments and analyzed the data. JL, JJ, PL, and HL interpreted the results and wrote the manuscript. All the authors read and approved the final manuscript.

## Conflict of Interest Statement

The authors declare that the research was conducted in the absence of any commercial or financial relationships that could be construed as a potential conflict of interest.
